# Development of Effective Therapeutics Targeting HER3 for Cancer Treatment

**DOI:** 10.1186/s12575-019-0093-1

**Published:** 2019-03-19

**Authors:** Xiaolong Liu, Shuang Liu, Hui Lyu, Adam I. Riker, Yamin Zhang, Bolin Liu

**Affiliations:** 1 Department of Hepatobiliary Surgery, Tianjin First Central Hospital, Tianjin, China; 2 Department of Genetics, Stanley S. Scott Cancer Center, School of Medicine, Louisiana State University Health Sciences Center, New Orleans, LA USA; 3 Department of Surgery, Section of Surgical Oncology, Stanley S. Scott Cancer Center, School of Medicine, Louisiana State University Health Sciences Center, New Orleans, LA USA

**Keywords:** HER3, Cell signaling, Targeted therapy, Epigenetic approach, miRNA

## Abstract

HER3 is the third member of the human epidermal growth factor receptor (HER/EGFR) family, and unlike its other family members, is unique due to its minimal intrinsic kinase activity. As a result, HER3 has to interact with another receptor tyrosine kinase (RTK), such as EGFR or HER2, in order to activate the PI-3 K/Akt, MEK/MAPK, Jak/Stat pathways, as well as Src kinase. Over-expression of HER3 in various human cancers promotes tumor progression by increasing metastatic potential and acting as a major cause of treatment failure. Effective inhibition of HER3, and/or the key downstream mediators of HER3 signaling, is thought to be required to overcome resistance and enhance therapeutic efficacy. To date, there is no known HER3-targeted therapy that is approved for breast cancer, with a number of anti-HER3 antibodies current in various stages of development and clinical testing. Recent data suggests that the epigenetic strategy of using a histone deacetylase (HDAC) inhibitor, or functional cooperative miRNAs, may be an effective way to abrogate HER3 signaling. Here, we summarize the latest advances in our understanding of the mechanism of HER3 signaling in tumor progression, with continuing research towards the identification of therapeutic anti-HER3 antibodies. We will also examine the potential to develop novel epigenetic approaches that specifically target the HER3 receptor, along with important key downstream mediators that are involved in cancer treatment.

## Introduction

The human epidermal growth factor receptor (HER) family, including the epidermal growth factor receptor (EGFR, or HER1/erbB1), HER2 (or erbB2/*neu*), HER3 (or erbB3), and HER4 (erbB4) is arguably the most important receptor tyrosine kinase (RTK) family involved with normal cell development and tumorigenesis [1, 2]. Elevated expression of the HER family members is frequently observed in a wide variety of human cancers, including colorectal cancer, gastric cancer, breast cancer, non-small cell lung cancer (NSCLC), ovarian cancer, head and neck cancer, pancreatic cancer and cervical cancer, and has been shown to play a critical role in cancer development [3, 4]. Both EGFR and HER4 have several ligands, with HER3 exhibiting only a single ligand, called heregulin (HRG) or neuregulin (NRG). The HER2 receptor has no known ligand. When a ligand binds to the extracellular region of EGFR, HER3, or HER4, it leads to a receptor-receptor interaction that results in dimerization [5], which is one of the key features of the HER receptors having the capacity for any two of the family members to form either homo- or hetero-dimers. Dimerization is the initial step critical for HER receptor function and activation of the downstream signaling, such as the PI-3 K/Akt, MEK/MAPK, Jak/Stat pathways, Src kinase, and several others [4, 6]. EGFR, HER3, and HER4 normally exist as molecularly folded monomers (inactive state) to prevent dimerization [7, 8]. In contrast, HER2 receptor always stays in a constitutively active conformation with its dimerization arm opening ready for interaction with another receptor [7].

Unlike other HER family members (EGFR, HER2, and HER4), the HER3 receptor has little or no tyrosine kinase activity [9, 10]. It has been shown that HER3 frequently co-expresses, and interacts with, another RTK to form a heterodimeric complex, which subsequently activates oncogenic signaling. This is especially true for the PI-3 K/Akt pathway and Src kinase to increase cancer cell survival and proliferation [11–13]. Studies on the underlying mechanisms demonstrate that HER3 signaling promotes cancer progression mainly through influencing two aspects of cancer biology, mainly by enhancing metastatic potential of tumor cells and causing treatment failures in cancer therapy [14–16]. There is accumulating data that strongly support the notion that developing effective HER3-targeted therapy is required to overcome resistance, enhance treatment efficacy and increase survival rates of cancer patients. Due to the lack of, or weak, kinase activity [9, 10], targeting HER3 with a blocking antibody (Ab) is the only strategy currently being examined in pre-clinical studies and clinical evaluation in cancer patients [17–21]. Recent studies offer new hopes to develop epigenetic approaches, such as using a histone deacetylase inhibitor (HDACi) [22, 23] specific miRNAs [24–26], or by targeting HER3 and its key downstream mediators.

### HER3 Promotion of Tumor Metastasis and Function as a Major Determinant of Cancer Drug Sensitivity

Elevated expression of HER3 is frequently observed in a variety of human cancers, with its over-expression of HER3 associated with poor clinical outcomes [12, 27–32]. HER3 must interact with another receptor to transduce cell signaling, often partnering with HER2 to exhibit its oncogenic activity in tumors with HER2 over-expression [12, 33–35]. A recent report further demonstrates that over-expression of HER3 is associated with a poorer survival rate in patients with cancers, including colorectal cancer, gastric cancer, breast cancer, melanoma, ovarian cancer, head and neck cancer, pancreatic cancer and cervical cancer [36]. Moreover, it showed that the impact of HER3 on clinical outcomes is much more prevalent in the tumors simultaneously over-expressing HER2 [36]. This suggested that it is the HER2/HER3 heterodimer that plays a crucial role in cancer progression. Others have shown that enhanced HER3 signaling facilitates tumor cell motility and intravasation in metastatic breast cancer to the lung [37]. It was identified that a HER3-lncRNA (long non-coding RNA) axis regulates bone metastasis in breast cancer [38, 39].

In addition, the HER3 ligand, HRG, can stimulate chemotaxis and invasion via HER2/HER3 heterodimers [40]. It has been reported that HRG-induced activation of HER3 signaling is important in breast cancer brain metastasis [41, 42]. In addition to brain metastases derived from a primary breast cancer, which can overexpress HER3 [42, 43], increased HRG production by the stromal cells within the brain microenvironment may also result in activation of HER3 and its downstream signaling, thereby promoting breast cancer brain metastasis [41, 42, 44]. Activation of the PI-3 K/Akt and MEK/MAPK signaling, two major downstream pathways of HER3 signaling, can be critical for cell motility and chemotaxis [40, 45–49]. The PI-3 K pathway is able to regulate cytoskeleton and cancer cell survival through Rho family G proteins and Akt activation, respectively [50–52]. The MAPK’s control cell proliferation, adhesion, and gene expression essential for motility and invasion [53–55]. It is possible that HER3-dependent motility contributes to cancer metastasis independent of its effects on tumor growth [37]. A recent study challenges our current view on tumor metastasis of ovarian cancer. While local spread to the omentum was thought to be the main mechanism of ovarian cancer metastasis, it shows that elevated expression of HER3 in ovarian cancer cells and increased HRG in the omentum allows for cancer cell localization and growth in the omentum. In fact, HRG-induced HER3 signaling appears to be the dominant pathway involved with the hematogenous metastasis of ovarian cancer [56].

Interestingly, non-coding RNA (ncRNA), such as the long ncRNA (lncRNA) *MAYA* has also been shown to play an important role in HER3-mediated tumor metastasis [39]. Upon HRG stimulation, the RTK-like orphan receptor ROR1 phosphorylates HER3. The phosphorylated HER3 then recruits a *MAYA*-containing RNA-protein complex to methylate Hippo/MST1. This methylation further leads to MST1 inactivation and activation of YAP target genes in breast cancer cells, thereby inducing osteoclast differentiation and bone metastasis [38]. Thus, the ROR1-HER3-lncRNA (*MAYA*) axis represents a novel mechanism regulating the Hippo-YAP pathway to control bone metastasis in breast cancer [38, 39]. In the last several years, our laboratory has strived to identify key downstream mediators of HER3 signaling in metastatic breast cancer. Two tumor suppressive miRNAs, miR-203 and miR-542-3p were found to be specifically down-regulated by HER3 signaling in HER2-over-expressing breast cancer cells [25]. Further analyses reveal that both miR-203 and miR-542-3p target a cohort of genes, including *Survivin, ZEB1*, *ZEB2*, and *Snail1*, responsible for drug resistance, epithelial-mesenchymal transition (EMT) and tumor metastasis (Liu lab unpublished data). Our data suggest that HER3 signaling may promote cancer metastasis via modulating expression of specific miRNAs. We believe that such studies in this innovative area will provide a new avenue for the identification of novel therapeutic approaches to abrogate HER3-mediated cancer metastasis.

Numerous studies implicate HER3 activation as a major cause of treatment failure in cancer therapy [15]. HER3 signaling plays a crucial role in the development of human cancers that exhibit a drug resistance phenotype, including HER2-over-expressing breast cancer [11, 12], castration-resistant prostate cancer [57], platinum-resistant/refractory ovarian cancer [58, 59], and EGFR tyrosine kinase inhibitor (TKI)-resistant non-small cell lung cancer (NSCLC) [60, 61]. It is now clear that the compensatory up-regulation of HER3 along with the sustained PI-3 K/Akt signaling is an important mechanism resulting in resistance to EGFR-targeted therapy, gefitinib [1, 62–64]. In addition, elevated expression of HRG has been shown to be a possible mechanism of resistance to the anti-EGFR Ab, cetuximab, in patients with colorectal cancer [65]. For squamous cell carcinoma of the head and neck, cell lines sensitive to the dual EGFR/HER2 inhibitor, lapatinib, increased HRG and strongly activated HER3 which correlated with lapatinib sensitivity [66].

However, the potential mechanism by which HER3 may be a valuable biomarker for lapatinib sensitivity and gefitinib resistance remains unclear. It may be through distinct activation mechanisms that need to be further investigated. Our laboratory has been focusing on understanding the biologic function of HER3 as it relates to the progression of HER2-over-expressing breast cancer. We also show that elevated expression of HER3 in HER2-over-expressing breast cancer cells results in resistance to hormone therapy (tamoxifen), HER2-targeted therapy (trastuzumab and lapatinib) and chemotherapy (paclitaxel) [67–71]. Our data demonstrate the crucial role of HER3 signaling in HER2-mediated therapeutic resistance in breast cancer [13, 16]. One interesting observation arises from our studies on the underlying mechanism of HER3-mediated resistance to trastuzumab (or Herceptin). While both HER3 and the insulin-like growth factor-I receptor (IGF-1R)-mediated signaling have been reported to contribute to trastuzumab resistance [72–74], the relationship between HER3 and IGF-IR in trastuzumab resistance was not previously appreciated.

We found that HER2 interacted with both HER3 and IGF-1R, forming a heterotrimeric complex in trastuzumab-resistant breast cancer cells. In fact, it was the heterotrimer of HER2/HER3/IGF-1R that played a causal role leading to trastuzumab resistance [67]. Further studies revealed that HER3 and IGF-1R triggered different signaling pathways contributing to trastuzumab resistance, with HER3 activating both PI-3 K/Akt signaling and Src kinase, whereas IGF-1R mainly influenced Src activation [67]. Interestingly, our recent data shows that HER3 and IGF-1R exhibit distinct effects upon the sensitivity of HER2-over-expressing breast cancer cells to lapatinib [71]. While HER3 signaling also induces lapatinib resistance in the trastuzumab-resistant breast cancer cells, IGF-1R signaling did not alter lapatinib sensitivity [71]. Our studies on the molecular mechanism of HER3-mediated resistance to chemotherapy paclitaxel showed that survivin, up-regulated by HER3, served as a key downstream mediator in HER3 signaling-induced paclitaxel resistance [70].

### Therapeutic Antibodies against HER3 in Clinical and Pre-Clinical Investigations

The elevated expression of HER3 promotes cancer progression and correlates with a worse survival rate in patients with cancers of colon, gastric, breast, lung, ovarian, melanoma, head and neck, pancreatic and cervical [15, 36, 75], emphasizing the importance of developing effective therapeutics that specifically target and inhibit the HER3 receptor [16, 76, 77]. It is believed that inactivation of HER3, as well as its downstream signaling, is required to overcome this resistance and effectively treat cancer patients. Due to the intrinsic low kinase activity [9, 10], targeting HER3 with a blocking Ab has been the only strategy examined in pre-clinical studies [78, 79] and for patients with advanced solid tumors (http://www.clinicaltrials.gov). Advances have been made to identify HER3-targeted therapy [17, 80, 81], and a number of anti-HER3 Abs exhibit anti-tumor activity in vivo and show promise as novel cancer therapeutics [18, 82, 83]. In addition to developing monoclonal Abs directly against HER3, recent studies have also identified bi-specific Abs that are dual targets for EGFR/HER3 [61] or HER2/HER3 [84]. These exert potent anti-tumor activities in both laboratory studies and clinic investigations [80]. As this review focuses on human cancers, Table 1 only lists the current clinical trials in cancer patients to test the therapeutic activity of several anti-HER3 monoclonal and bi-specific Abs.

**Table 1:** Mono- and bi-specific anti-HER3 Abs under clinical studies in cancer patients

Abs	Target	Most advanced clinical phase	Clinicaltrials.gov identifier	Current Results on clinicaltrials.gov	Sponsor
mAbs:					
U3–1287/Patritumab	HER3	Phase III	NCT02134015	Terminated (Pre-defined criteria Not reached)	Daiichi Sankyo
MM-121/Seribantumab	HER3	Phase II	NCT00994123	MM-121+ erlotinib ineffective to prolong PFS in EGFR WT NSCLC	Merrimack Pharmaceuticals
RG7116/Lumretuzumab	HER3	Phase I	NCT01482377	No results posted	Roche
LJM716/Elgemtumab	HER3	Phase I/II	NCT01822613	No results posted	Novartis
U3–1402	HER3	Phase I/II	NCT02980341	Ongoing	Daiichi Sankyo
AV-203	HER3	Phase I	NCT01603979	No results posted	Aveo Oncologyo
KTN3379/CDX-3379	HER3	Phase I	NCT02014909	No results posted	Celldex Therapeutics
GSK2849330	HER3	Phase I	NCT01966445	Results submitted, But not posted	GlaxoSmithKline
Bispecific Abs:					
MM-111	HER2/HER3	Phase II	NCT01774851	Terminated (Lack of efficacy)	Merrimack Pharmaceuticals
MCLA-128	HER2/HER3	Phase II	NCT03321981	Ongoing	Merus NV
MM-141/Istiratumab	HER3/IGF-1R	Phase II	NCT02399137	No results posted	Merrimack Pharmaceuticals
MEHD7945A/Duligotumab	HER3/EGFR	Phase II	NCT01652482	No results posted	Genentech

The development of U3–1287/AMG-888 (originally developed by Amgen Inc., Thousand Oaks, CA; later acquired by Daiichi Sankyo Co. Ltd., Tokyo, Japan and re-named as patritumab) is the first fully humanized, anti-HER3 monoclonal Ab that is currently being examined in several clinical trials in patients with advanced solid tumors [18], including a phase III trial in patients with NSCLC [85]. This Ab was able to inhibit proximal and distal HER signaling and induce rapid internalization of HER3 [86]. The Ab, patritumab, inhibited cell proliferation in various cancer cell lines (breast, lung, colorectal) that are resistant to other HER inhibitors [86]. It dramatically reduced colony formation of pancreatic cancer cells and inhibited tumor growth in tumor xenograft models of pancreatic cancer, NSCLC and colorectal cancer [57]. Patritumab has been shown to overcome HRG-dependent resistance to EGFR inhibitors in NSCLC in vitro and in vivo*.* Such data further supports the interest in ongoing clinical trials testing patritumab in combination with EGFR TKIs, such as erlotinib, to treat NSCLC patients with high expression of HRG [85, 87, 88].

The monoclonal Ab, MM-121/seribantumab (Merrimack Pharmaceuticals, Cambridge, MA), is a human, anti-HER3 monoclonal IgG2 Ab. It blocks ligand-induced HER2/HER3 dimerization and inhibits downstream signaling. MM-121 exerts potent anti-tumor activity in pre-clinical studies of various human cancers [78, 79]. We have shown that MM-121 was able to abrogate HER3 signaling-mediated resistance to trastuzumab and paclitaxel in HER2-over-expressing breast cancer cells via the inactivation of HER3 and its downstream PI-3 K/Akt signaling [89, 90]. Our data may facilitate the development of clinical trials to test the efficacy of MM-121 in combination with trastuzumab or paclitaxel in HER2-overexpressing breast cancer patients who have developed resistance to trastuzumab or paclitaxel.

Interestingly, recent studies suggest that higher HRG mRNA expression, and low HER2 levels predict a clinical benefit from the addition of seribantumab (MM-121) to standard of care therapies in patients with platinum-resistant/refractory ovarian cancer, hormone receptor-positive HER2-low breast cancer and EGFR wild-type NSCLC [91, 92]. Lumretuzumab/RG7116 (Roche Diagnostics GmbH, Penzberg, Germany) is a humanized anti-HER3 IgG1 monoclonal Ab. It binds to the extracellular domain of HER3 with high affinity to prevent HRG binding [93]. As a glyco-engineered Ab, lumretuzumab has an enhanced antibody-dependent cell-mediated cytotoxicity (ADCC) activity when compared with the non-glyco-engineered parental antibody [17]. Although lumretuzumab was well tolerated and showed evidence of clinical activity in a phase I trial [19], two recent phase Ib studies suggest otherwise. The toxicity profile of lumretuzumab in combination with the EGFR-targeted therapies, cetuximab and erlotinib, was manageable, but it exerted only a minimal clinical benefit in various cancers [20]. The therapeutic window of lumretuzumab in combination with the anti-HER2 Ab pertuzumab and chemotherapeutic drug paclitaxel for HER3-positive metastatic breast cancer was too narrow to warrant further clinical development [21]. MM-111 (Merrimack Pharmaceuticals, Cambridge, MA) is a bi-specific Ab, dual-targeting HER2/HER3, inhibiting the PI-3 K/Akt signaling [84]. The safety and clinical activity of MM-111 is now being tested in several phase I/II clinical trials of cancer patients [81, 85].

It is worth mentioning that the anti-HER3 Ab (MP-RM-1), and its humanized version, (EV20) exhibit potent anti-tumor effects in several cancer types in vitro and in vivo [94, 95]. Although EV20 is only being examined in the pre-clinical setting, its capacity to inhibit both ligand-dependent and independent activation of HER3 [94, 95] yields a high level of enthusiasm that EV20 may have a broader effect on blocking HER3 signaling compared with other similar Abs (like MM-121) that only block ligand-induced HER3 activation. In addition to developing specific Abs directly against HER3, recent studies have attempted to identify Ab-like agent(s) targeting HER3. The HER3 inhibitors are based upon a novel biologic scaffold, termed surrobody, that has been developed, showing significant anti-proliferative effects on cancer cells in vitro and in vivo [96].

An HER1–3-neutralizing Ab mixture exerts a high antitumor activity against drug-resistant HER2-overexpressing breast cancers, suggesting that the multi-targeted Ab mixture represents a novel approach for effective treatment of breast cancers with HER2-overexpressing tumors [97]. Interestingly, an adenovirus encoding the full length human HER3 (Ad-HER3) receptor was generated to be utilized as a putative cancer “vaccine” [98]. Ad-HER3 not only induced potent T-cell anti-tumor responses, the HER3, vaccine-induced antibodies (HER3-VIAs) also provided additional activity to eliminate tumors in which HER3 signaling mediates aggressive behavior or acquired resistance to HER2-targeted therapy and triple-negative breast cancers [98]. Thus, clinical studies of vaccination against HER3 in combination with other therapies, such as trastuzumab to treat the refractory HER2-overexpressing breast cancers or chemotherapy like paclitaxel against triple-negative breast cancers may show a plausible therapeutic efficacy.

### Emerging Strategy Targeting of HER3 Signaling with Therapeutic Potential

**Fig. 1:**
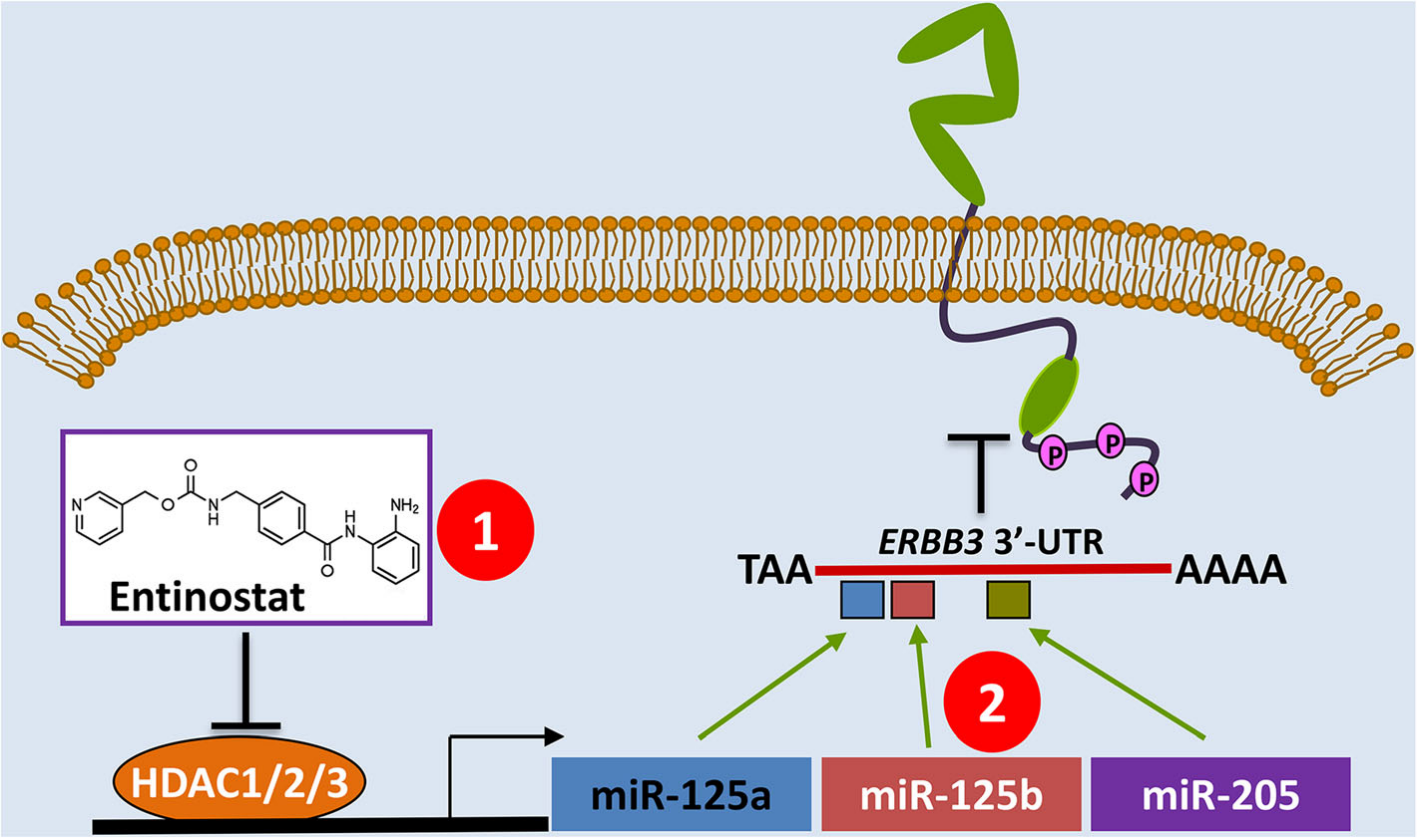
A diagram showing the novel epigenetic approaches inhibiting HER3 for cancer treatment. The class I HDACi entinostat potently downregulates HER3 expression via induction of miR-125a, miR-125b, and miR-205, which act in concert to inhibit HER3 protein translation. Thus, the epigenetic strategy takes advantage of a novel mechanism of action (distinct from that of an anti-HER3 Ab) to abrogate HER3 signaling

There has been a lot of research effort put forth to identify novel therapeutics and approaches which can effectively inhibit HER3, or key downstream mediators of HER3 signaling. To this end, we discovered that the class I HDACi, entinostat, was able to potently down-regulate HER3 in HER2-over-expressing breast cancer cells [22]. Further studies revealed that entinostat induced expression of miR-125a, miR-125b, and miR-205, all of which were reported to directly target the 3’UTR of *erbB3* mRNA [99, 100]. The three miRNAs acted in concert to inhibit HER3 protein translation in HER2-over-expressing breast cancer cells [101]. Such exciting data support a novel hypothesis that effective targeting of HER3 may be achieved with the treatment of entinostat, or with functional cooperation from miR-125a, miR-125b, and miR-205 (Fig. 1).

Thus, we examined this idea with miR-125a and miR-205 in order to determine whether co-expression of the two miRNAs would exert functional cooperation to inhibit *erbB3* expression in HER2-over-expressing breast cancer cells. Our data showed that the miRNA (miR-125a/miR-205) cluster (co-expression of miR-125a and miR-205) was more effective than either miRNA alone to down-regulate HER3 in HER2-over-expressing breast cancer cells [24]. Importantly, we discovered that the miRNA (miR-125a/miR-205) cluster not only profoundly inhibited cell proliferation, but also significantly enhance trastuzumab- and paclitaxel-mediated anti-proliferative/anti-survival effects on HER2-over-expressing breast cancer cells [24]. Our data strongly support the notion that the miR-125a/miR-205 cluster may be developed as a novel, effective HER3-targeted therapy to enhance therapeutic efficacy against HER2-over-expressing breast cancer.

Identification of the crucial downstream mediator(s) that relay(s) HER3 signaling-induced resistance shall facilitate the development of novel approaches to inhibit HER3 signaling and thereby enhance therapeutic efficacy for cancer treatment. Our previous studies found that elevated expression of HER3 conferred paclitaxel resistance in HER2-over-expressing breast cancer cells via up-regulation of survivin [70]. Although survivin has long been considered as a good molecular target for cancer treatment [102, 103], there has been no survivin-targeted therapy to date, with currently available strategies lacking both specificity and effectiveness [104]. We have recently shown that miR-203 and miR-542-3p play an essential role in HER3/PI-3 K/Akt signaling-mediated up-regulation of survivin [25]. These data provided an opportunity to examine miRNA-based therapeutic strategy inhibiting survivin to overcome HER3-mediated paclitaxel resistance. In order to define whether miR-203 and miR-542-3p may be useful for survivin-targeted therapeutics, we first performed bioinformatics analyses and found that miR-542-3p has three binding sites on the 3′-UTR of survivin mRNA, whereas miR-203 has only one binding site [105].

This interesting observation inspired us to test the hypothesis that the miRNAs with multiple binding sites on the 3′-UTR of survivin mRNA shall be more effective than those with a single binding site in the down-regulation of survivin. Both in vitro and in vivo experiments revealed that introduction of miR-542-3p mimic not only exhibited a more potent activity to specifically down-regulate survivin, but also markedly enhanced paclitaxel-mediated anti-tumor effects via inhibition of proliferation and induction of apoptosis [25]. These data suggest that miR-542-3p-replacement therapy holds potential for further development as a novel strategy for surviving inhibition, thereby overcoming HER3 signaling-induced paclitaxel resistance.

Moreover, our study further support the idea that functional cooperation exists among the multiple binding sites of one miRNA, which is in agreement with our recent report showing that the miR-125a/miR-205 cluster potently inhibits HER3 expression in HER2-over-expressing breast cancer cells [24]. It is likely that the multiple binding sites of one miRNA and the “sister” miRNAs, which have common targets [26], act synergistically to repress the target, suggesting that the miRNAs with multiple binding sites may be more promising in miRNA-replacement therapy. Thus, a novel epigenetic strategy has emerged to target HER3 and/or its key downstream mediator to abrogate HER3-mediated treatment failure in cancer therapy. The HDACi entinostat or the miR-125a/miR-205 cluster inhibit HER3 expression and miR-542-3p may act as an effective survivin-targeted therapy, all of which will overcome HER3 signaling-mediated resistance and thereby enhance therapeutic efficacy against HER2-overexpressing breast cancer. Due to the novel epigenetic approach that aims to reduce HER3 or survivin protein levels, not just inhibits HER3 signaling, it has great potential to eliminate the chance for tumor cells to develop resistance after an initial response to standard therapy.

### Future Development

Clearly, a therapy that can effectively inhibit HER3 signaling is required to overcome drug resistance, enhance therapeutic efficacy and increase survival of cancer patients. While several anti-HER3 Abs with therapeutic potential are actively under clinical evaluations, the hope is high for a select few, mainly MM-121/seribantumab and patritumab, both of which have shown encouraging clinic benefits in patients with non-small cell lung cancer [88, 92]. In addition, the US FDA has recently (10/30/2017) granted an orphan drug designation to MM-121 for the treatment of HRG-positive NSCLC (http://investors.merrimack.com/node/11346). Prior to its final approval, one of the obstacles may have to attribute to the unique biologic feature of the HER3 receptor. As we know, HER3 has to interact with, and usually acts as a co-receptor for, another RTK, with HER2 being the most important one [106]. Thus, therapeutic targeting of HER3 alone may not show dramatic anti-tumor effects. It needs to combine with other treatment(s). The challenges are directed towards a HER3-targeted therapy combined with a second effective therapy, and how to combine a HER3-targeted therapy with other therapeutics. Lastly, it is a relevant question to ask when to combine a HER3-targeted therapy together with another agent. Further investigations on these questions should provide valuable data to facilitate the FDA approval for the anti-HER3 Abs currently under clinical testing.

The emerging epigenetic approaches targeting HER3 and/or its signaling are theoretically based upon RNA interference (RNAi) technology-based therapy, such as “small interfering RNA” (siRNA)- or miRNA-replacement therapy, which is actively being explored as a new strategy to treat human diseases, including cancer [107]. The regulatory potential of miRNAs on the entire signaling networks within the cells, and involvement in cancer development and progression, has resulted in the miRNAs as promising molecular targets for cancer treatment [108–111]. Recent studies in this area have driven the future development of miRNAs as cancer therapeutics, moving very quickly from the bench to clinic application [107, 112, 113]. Unfortunately, the first clinical trial using miR-34 mimics (trade name: MRX34, Mirna Therapeutics, Austin, TX) as a replacement therapy, failed due to multiple immune-related side effects. It was hoped that further analysis of its full pre-clinical and clinical data would provide useful information on the future development of MRX34 as a cancer therapeutics (https://www.bizjournals.com/austin/news/2016/09/21/austin-drug-company-halts-clinical-studies-after.html). In our effort to identify novel therapeutic approach targeting HER3, we find that the miR-125a/miR-205 cluster potently inhibits HER3 expression [24], and miR-542-3p, because of its three binding sites on the 3’UTR of survivin mRNA, and holds great potential as an effective survivin-targeted therapy [25]. Our data strongly suggest that in the future, functional cooperative miRNAs or the miRNAs with multiple binding sites on a target may be more promising candidates for the development of miRNA-replacement therapy. A few months ago (8/10/2018), the US FDA approved the first-ever, siRNA product as an Orphan Drug Designation (Onpattro or patisiran) to treat the rare hereditary disease transthyretin-mediated amyloidosis in adult patients (https://www.biologicsblog.com/fda-approves-first-ever-sirna-therapy). This approval marks a significant milestone in the story of RNAi technology and clearing the way for a new type of therapeutic strategy. We believe that the novel epigenetic approaches, using a specific HDACi (entonostat) or miRNA-replacement therapy, targeting HER3 and/or its key downstream mediator deserves further investigation for cancer treatment.

## Abbreviations

Ab: Antibody
EGFR: Epidermal growth factor receptor
EMT: Epithelial-mesenchymal transition
FDA: Food and Drug Administration
HDAC: Histone deacetylase
HDACi: HDAC inhibitor
HER: Human epidermal growth factor receptor
HRG: Heregulin
IGF-1R: Insulin-like growth factor-I receptor
lncRNA: Long ncRNA
MAPK: Mitogen-activated protein kinase
MEK: MAPK kinase
miRNA: microRNA
ncRNA: Noncoding RNA
NSCLC: Non-small cell lung cancer
PI-3 K: Phosphoinositide 3-kinase
RTK: Receptor tyrosine kinase
TKI: Tyrosine kinase inhibitor
